# First Functional and Mutational Analysis of Group 3 N-Acetylneuraminate Lyases from *Lactobacillus antri* and *Lactobacillus sakei 23K*

**DOI:** 10.1371/journal.pone.0096976

**Published:** 2014-05-09

**Authors:** María Inmaculada García-García, Fernando Gil-Ortiz, Francisco García-Carmona, Álvaro Sánchez-Ferrer

**Affiliations:** 1 Department of Biochemistry and Molecular Biology-A, Faculty of Biology, Regional Campus of International Excellence “*Campus Mare Nostrum*”, University of Murcia, Campus Espinardo, Espinardo, Murcia, Spain; 2 Murcia Biomedical Research Institute (IMIB), El Palmar, Murcia, Spain; 3 CELLS-ALBA Synchrotron Light Source, Cerdanyola del Vallès, Barcelona, Spain; Institute of Enzymology of the Hungarian Academy of Science, Hungary

## Abstract

N-acetyl neuraminate lyases (NALs) catalyze the reversible aldol cleavage of N-acetyl neuraminic acid (Neu5Ac) to pyruvate and N-acetyl-D-mannosamine (ManNAc). Previous phylogenetic studies divided NALs into four different groups. Groups 1 and 2 have been well characterized at both kinetic and molecular levels, but no NAL from group 3 has been studied to date. In this work, a functional characterization of two group 3 members was performed using the recombinant NALs from *Lactobacillus antri* and *Lactobacillus sakei* 23K, revealing an optimal pH of between 6.0 and 7.0, low stability at basic pHs (>8.0), low optimal temperatures and, especially, low catalytic efficiency compared with their counterparts in group 1 and 2. The mutational analysis carried out showed that a plausible molecular reason for the low activity shown by *Lactobacillus antri* and *Lactobacillus sakei* 23k NALs compared with group 1 and 2 NALs could be the relatively small sugar-binding pocket they contain. A functional divergence analysis concluding that group 3 is more closely related to group 2 than to group 1.

## Introduction

Sialic acids or nonulosominic acids are a family of nine-carbon amino sugars found at the terminal positions of glycoproteins and glycolipids [1]. In the human body these essential cell-surface residues are associated with inflammatory diseases, cancer metastasis, and influenza virus infection [2]. Among the more than 50 naturally occurring sialic acids found in both eukaryotes and prokaryotes, N-acetyl-D-neuraminic acid (2-keto-3-deoxy-5-acetamino-D-glycero-D-galacto-nonulosonic acid or Neu5Ac) is the most abundant and widely studied [3]. Several pathogenic bacteria use this sialic acid to mask themselves from the host immune system, transferring these amino sugars to their outer surface by mean of different mechanisms that include *de novo* biosynthesis, sialic acid scavenging or precursor scavenging [4]. On the other hand, bacteria can also use Neu5Ac as a carbon and nitrogen source by scavenging it from the mucus-rich environment [5]. The first step of this catabolic pathway is catalyzed by a pyruvate-dependent lyase, N-acetylneuraminate lyase (NAL; EC 4.1.3.3) or Neu5Ac aldolase. This enzyme cleaves Neu5Ac into pyruvate and N-acetyl mannosamine (ManNAc), which is then, either phosphorylated to ManNAc-6P and later epimerized to N-acetylglucosamine-6-phosphate (GlcNAc-6-P), or first epimerized to GlcNAc and then phosphorylated to GlcNAc-6-P [6]. It is this latter compound that enters the common pathways of amino sugar utilization [6]. This sialic acid catabolism process has been found in *Clostridium perfringens* [7], *Escherichia coli* [8], *Pasteurella multocida* [9], *Haemophilus influenza* [10], *Bacteroides fragilis* [11] and, recently, in *Lactobacillus sakei* 23K [12]. However, enzymes related with such catabolism have been described in more microorganisms, including *Lactobacillus plantarum* [13], *Staphylococcus carnosus* [14] and *Bacteroides ovatus* [15].

In fact, genes encoding NAL (*nanA* gene) are limited to human commensal and pathogenic bacteria and a few aquatic bacteria (*Photobacterium profundum*, *Pseudomonas haloplanktis, Shewanella pealeana, Psychromonas,* and *Vibrio*) [5]. Based on phylogenetic analysis and on the structural blocks of the NAL active site, four structural groups have been described [13]. The first NAL group has been extensively characterized using two enzymes from Gram- bacteria, *H. influenzae* [10] and *C. perfringens* [7] and two from Gram+ bacteria, *L. plantarum* [13] and *S. carnosus* [14]. Group 2 includes the enzyme from *E. coli* [8], [16], which represents the model for this group, since the remaining members share high sequence identity (∼90–100%) [13]. So far, no NALs from group 3 or group 4 have been characterized. Interestingly, group 3, while related to group 1 and group 2, has its own fully conserved active center signature, which also differs from the diversity shown by group 4, which displays four different subgroups (4.1 to 4.4) [13].

To elucidate the underlying biochemical and molecular basis of this NAL classification, this paper describes the cloning, overexpression and detailed characterization of the N-acetylneuraminate lyase gene (*nanA*) from *Lactobacillus antri* (LaNAL). The enzyme showed clear biochemical differences from group 1 and 2 enzymes, including an optimal pH close to 7, low stability at basic pH, low temperature stability and low catalytic efficiency. To answer the question whether or not these features were also present in other group 3 NAL members, *Lactobacillus sakei 23K* NAL (LsNAL) was also cloned and characterized, and similar results were obtained. A detailed functional study through the directed mutagenesis of key residues has allowed us to identity the likely reason for the differences encountered with NALs from groups 1 and 2.

## Materials and Methods

### Bacterial strains and chemicals

*L. antri* was from the DSMZ collection (#16041) and the genomic DNA from *L. sakei 23K* was kindly provided by Prof. Monique Zagorec (Unité Flore Lactique et Environnement Carné, INRA, France). *N*-acetyl-D-mannosamine, *N*-acetylneuraminic acid and other sugars were from Carbosynth (Berkshire, UK). ManNAc dehydrogenase was from Kikkoman (Japan, Tokio). Other reagents were from Sigma-Aldrich (Madrid, Spain).

### Cloning of LaNAL and LsNAL genes

The cloning and transformation techniques used were essentially those described by Sambrook et al [17]. *L. antri nanA* gene (828 bp) was amplified by PCR using the forward primer 5′CGCGCTAGCATGAAAGATTTTTCAAAGTATCG3′ and reverse primer 5′TATATCTCGAGCTAGTTGAATGCGGCG3′, which introduce *NheI-HF* and *Xho*I restriction sites. The corresponding *nanA* gene from *L. sakei 23K* (918 bp) was also amplified by PCR, but using the forward primer 5′*GCC* GCTAGCATGAAGGATTTAACGAAGTATAAAGGTA3′ and reverse primer 5′*CG*CCTCGAGCTAGCAATATTTTTCAATTGCA3′, which introduce *NheI-HF* and *Xho*I sites as above, respectively. The resulting PCR products were purified and digested with *Nhe*I-HF and *Xho*I restriction enzymes, ligated into the same sites of a predigested pET-28a vector (Merck Bioscience, Madison, WI, USA) and transformed into electrocompetent *E. coli* DH5α cells. A selected clone containing the pET28-*LaNAL* and pET28-*LsNAL* plasmids was isolated, sequenced, and transformed into *E. coli* Rosetta 2 competent cells (Merck Biosciences).

### Enzyme expression and purification

The *E. coli* Rosetta 2 cells harboring the recombinant plasmid pET28-*LaNAL* and pET28-*LsNAL* were grown for 4 hours at 37°C in 400 mL Luria-Bertani (LB) medium, containing kanamycin (50 µg mL^−1^) and chloramphenicol (34 µg mL^−1^) before being transferred to a 5-L fermenter (Sartorius), containing 4 L Terrific Broth supplemented with the same antibiotics. These cultures were allowed to grow for 3 h at 37°C, and then induced for 12 hours at 30°C with constant stirring and oxygenation by adding 1 mM isopropyl-β-D-thiogalactoside (IPTG) for LaNAL and 1.5 mM in the case of LsNAL. The cultures were diafiltered through a 500-kDa membrane (GE Life Sciences, Uppsala, Sweden) and cleaned with 50 mM phosphate buffer pH 8.0. Cells were disrupted using a bead homogenizer (MiniZetaII, Netzsch) and the cell debris was harvested by centrifugation. The recovered supernatant (crude extract) was treated with 3 U/mL DNase I (Sigma) to remove nucleic acids and then centrifuged for 20 min at 6000 *g.*

The purification in both cases was performed in two steps, starting with tangential ultrafiltration with a 100-kDa cutoff membrane on a QuixStand system (GE Healthcare). The resulting retentate was purified by Ni^2+^-chelating affinity chromatography (ÄKTA Prime Plus, GE Life Sciences) into a HiPrep column (GE Healthcare). The bound enzymes were eluted with a linear imidazole gradient up to 250 mM in 50 mM phosphate buffer pH 8.0. The fractions containing the aldolase activity were pooled, desalted, concentrated and stored at −20°C.

Protein concentrations were determined using Bradfords reagent (BioRad) [18] and bovine serum albumin as a standard. The molecular mass of the purified enzyme was determined by gel filtration (Superdex200 10/300 GL, GE Life Sciences) in 50 mM phosphate buffer pH 7.0, containing 0.15 M NaCl [13] or by HPLC/MS/ESI (Agilent Technologies), according to previously published methods [19]. The molecular mass under denaturing conditions (SDS-PAGE) was determined using 12% acrylamide gel.

### Enzyme activity assays

Neu5Ac enzymatic cleavage was determined both spectrophotometrically and chromatographically (HPLC). The first method measures the production of pyruvate by LaNAL or LsNAL, as a consequence of Neu5Ac cleavage, through the decrease in absorbance at 340 nm corresponding to the oxidation of NADH by lactate dehydrogenase (LDH) [14]. The standard reaction medium (1 mL) for the above assay, which was carried out in a Shimadzu UV-2401 PC spectrophotometer, contained 150 µM NADH, 0.5 U LDH, 10 mM Neu5Ac and 1 µg of purified LaNAL or 17 µg of purified LsNAL in 20 mM phosphate buffer pH 7.0. A control assay without Neu5Ac was also carried out in parallel to determine the presence of any other NADH-consuming enzymes. The hydrolytic activity was also measured from the increment of the ManNAc peak area, under the same reaction conditions using an HPLC-ELSD-II (Shimadzu, Duisburg, Germany), an Amino-UK column (Imtakt Co., Kyoto, Japan), and a mobile phase (58% acetonitrile: 42% 50 mM ammonium acetate) running at 0.4 mL/min at 60°C [13]. In these conditions, the retention time (R_T_) for Neu5Ac and ManNAc were 10.3 and 4.2 min, respectively. One unit of activity was defined as the amount of enzyme required to cleave 1 µmol of Neu5Ac, releasing 1 µmol of ManNAc in 1 min (HPLC) or consuming 1 µmol of NADH in 1 min at pH 7.0 and 37°C. The synthetic reaction was followed using the above HPLC conditions. The standard reaction medium for LaNAL contained 500 mM ManNAc, 10 mM pyruvate and 2 µg purified LaNAL in 20 mM phosphate buffer pH 7.0 or 500 mM ManNAc, 30 mM pyruvate and 50 µg purified LsNAL in the same buffer in the case of LsNAL. One enzymatic unit was defined as the amount of enzyme required to synthesize 1 µmol of Neu5Ac per minute under the above conditions.

Enzyme inhibition experiments for ManNAc were carried out spectrophotometrically using the above described reaction conditions but with different ManNAc (0–260 mM) and Neu5Ac (0.19–3.3 mM) concentrations. Enzyme inhibition by pyruvate was also measured spectrophotometrically at different pyruvate (0–5 mM) and Neu5Ac (0.19–3.3) concentrations, but using ManNAc dehydrogenase as a coupled enzyme (see below).

### Biochemical analysis

Substrate specificity tests of LaNAL with different sugars were carried out at 37°C with 0.6 M of each sugar, 1.2 M pyruvate and 1 mg/mL LaNAL in 20 mM sodium phosphate buffer pH 7.0. The samples were measured by HPLC-ELSD-II as described above, except for D-lyxose and 2-deoxy-D-glucose, in which the mobile phase was 70% acetonitrile: 30% 25 mM ammonium acetate.

The pH-stability assay was carried out by incubating the enzyme in the presence of 5 mM sodium pyruvate, or in its absence, at various pHs (6.0–9.0) at 37°C. The buffers used (20 mM) were sodium acetate pH 4.0–5.0, sodium phosphate pH 6.0–7.0, Tris-HCl pH 8.5 and glycine pH 9.0. Aliquots of 100 µL (1 µg LaNAL) were taken at different times, diluted 10-fold in the corresponding buffer to render a reaction medium containing 3 mM NAD+, 10 mM Neu5Ac and 10 U ManNAc dehydrogenase (EC: 1.1.1.233), and measured spectrophotometrically at 340 nm. This method measures the increase in absorbance corresponding to the reduction of NAD^+^ when the ManNAc produced by LaNAL is oxidized by the dehydrogenase into its corresponding lactone [15]. This method was used to avoid the interference caused by the sodium pyruvate added to the reaction and that generated by the enzyme in the presence of Neu5Ac. A heat-stability assay was carried out as described above but incubating the enzyme at different temperatures (60–80°C) using a PCR thermocycler (TGradient, Biometra, USA).

Temperature melting curves were determined using a commercial solution of SYPRO Orange (Molecular Probes) as previously described [13]. The assay was carried out in Milli-Q water or buffer containing 10X SYPRO Orange (emission at 530 nm and excitation at 490 nm), using a 7500 RT-PCR machine (Applied Biosystems). The time/temperature program was 70 steps of 1 min each, raising the temperature by 1°C steps, from 20°C to 90°C. This technique was also used to determine pH-stability.

### Site-directed mutagenesis

Two single-point mutants of LaNAL (G211S and P192Y) and a double mutant (G210S/Y213G) were constructed using the overlapping extension method [20]. The primers used for amplification are listed in Table S1. LaNAL double stranded plasmid DNA was extracted from *E. coli* DH5α cells and used as a template for the mutagenesis PCR. After amplification with Pfu Ultra II polymerase (Stratagene), PCR products were digested with *Dpn*I and transformed in *E. coli* DH5α electrocompetent cells. All mutations were confirmed by automated DNA sequencing.

### *In silico* analysis

Basic Local Alignment Search Tool (BLAST) searches were used to identify homologues of *N-*acetylneuraminate lyase [21] by using functionally characterized NALs from *H. influenzae, C. perfringens, L. plantarum* and *E. coli* K-12 (Uniprot codes: P44539, Q9S4K9, P59407 and P0A6L4, respectively). The sequences were aligned using ClustalW [22] and ESPript [23]. Automatic homology modeling was performed through the MODWEB modeling server (http://salilab.org/modweb) using the *E. coli* NAL structure as template (PDB ID 2WKJ). Superposition of the homology models was performed with the LSQ option of the COOT program [24]. The geometry of the theoretical models was refined using the idealization parameters of Refmac 55.6.0117 [25]. Functional divergence analysis was carried out using DIVERGE software [26]. Figures were drawn using Pymol (http://pymol.org/)

## Results and Discussion

### Homology analysis reveals low sequence identity with previously characterized NALs

**Figure 1:**
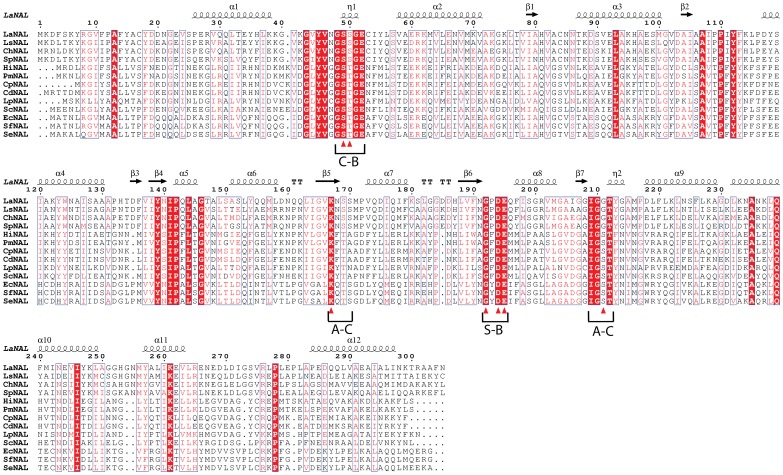
Multiple sequence alignment for LaNAL, LsNAL and related N-acetyl neuraminate lyases. ESPript outputs were obtained with sequences from SWISSPROT and aligned with CLUSTAL-W. Sequences were grouped according to similarity. Residues strictly conserved across NAL enzymes are highlighted against a red background. The secondary structure corresponding to the modeled LaNAL is shown, springs represent helices and arrows represent β-strands. The residues forming the active site are indicated with small triangles. The sequence motifs previously described (13) are C-B (carboxylate-binding motif), A-C (aldol-cleaveage motif) and S-B (sugar-binding motif). ChNAL, NAL from *Clostridium hylemonae*; SpNAL, NAL from *Streptococcus pneumoniae*; SfNAL, NAL from *Shigella flexneri*; SeNAL from *Salmonella enterica*.

Sequence alignment of LaNAL (UniProt code: C8P490) showed a 28% amino acid sequence identity with functionally characterized group 1 N-acetylneuraminate lyases (29% with *H. influenzae* NAL, *C. perfringens* NAL and *L. plantarum* NAL; 28% with *P. multocida* NAL and 26% with *S. carnosus* NAL), and 33% sequence identity with those of group 2 (33% with *E. coli* NAL, *Salmonella enterica* NAL and *Shigella flexneri* NAL) (Figure 1). Thus, LaNAL seems to be more closely related to crystallized EcNAL (PDB ID 1NAL) than to crystallized HiNAL (PDB ID 1F5Z). Other members of group 3, such as LsNAL, also showed an average sequence identity of 30% with group 1 and group 2 NALs (Figure 1).

Both group 3 NALs (LaNAL and LsNAL) showed the typical conserved residues of the NAL family at the active site (Figure 1, filled triangles), which consisted of a Schiff base forming catalytic lysine (K167, LaNAL numbering) inside a long conserved block (IGV**K**_167_NSSMPVQDIQ), a tyrosine (Y139) in block TDF [IV]I**Y**_139_NIPQLAG and a Neu5Ac binding motif, which included the carboxylate binding block (NG**S**_49_ **S**_50_GECIY) and the carbohydrate binding blocks (FN**G**_192_P**D**_194_ **E**_195_Q and IG**G**_211_TGYG).

### LaNAL and LsNAL are also tetrameric

The *L. antri nanA* gene was PCR-cloned into the pET-28a-derived (plasmid pET28-*LaNAL*), which labels the protein with N-ter His-tag. The expression was carried out in Terrific Broth medium at 30°C and 1 mM IPTG for 12 h with vigorous stirring and oxygenation in a 5-L fermenter. The crude protein was purified to homogeneity (Figure S1, lane 3) in only two-steps (see Materials and Methods): (i) 100-kDa ultrafiltration and (ii) Ni^2+^-chelating affinity chromatography, resulting in 3-fold purification with a 75% yield (Table S2). This recovery was 2 to 2.5-times higher than that described for other NALs, such as *L. plantarum* NAL (LpNAL), which was purified in three steps (42.3% recovery) [13], or *C. perfringens* NAL, which needed five steps to provide only a 21% recovery [27]. The purified enzyme showed a specific activity of 8.1 U mg^−1^, which was similar to that reported for the group 1 NALs such as LpNAL (7.65 U/mg) [13] but lower than that of ScNAL (12.7 U/mg) [14]. In addition, the yield of 157 mg litre^−1^ of initial culture in LaNAL was lower than that obtained for group 1 NALs (215–403 mg L^−1^) [13], [14]. *L. sakei 23K* NAL was cloned, expressed and purified as described for LaNAL, with a 2-fold purification, 51% recovery, specific activity of 1.5 U mg ^−1^ and a similar yield (151 mg L^−1^). The mass of both isolated proteins was determined by gel filtration (140 kDa), HPLC/MS/ESI (35.7 kDa for LaNAL, 33.5 kDa LsNAL) and SDS-PAGE (35 kDa LaNAL Figure S1, lane 3; and 35 kDa LsNAL (data not shown)), indicating their homotetrameric nature.

### LaNAL and LsNAL reveal low pH optimum and low thermal stability

**Figure 2:**
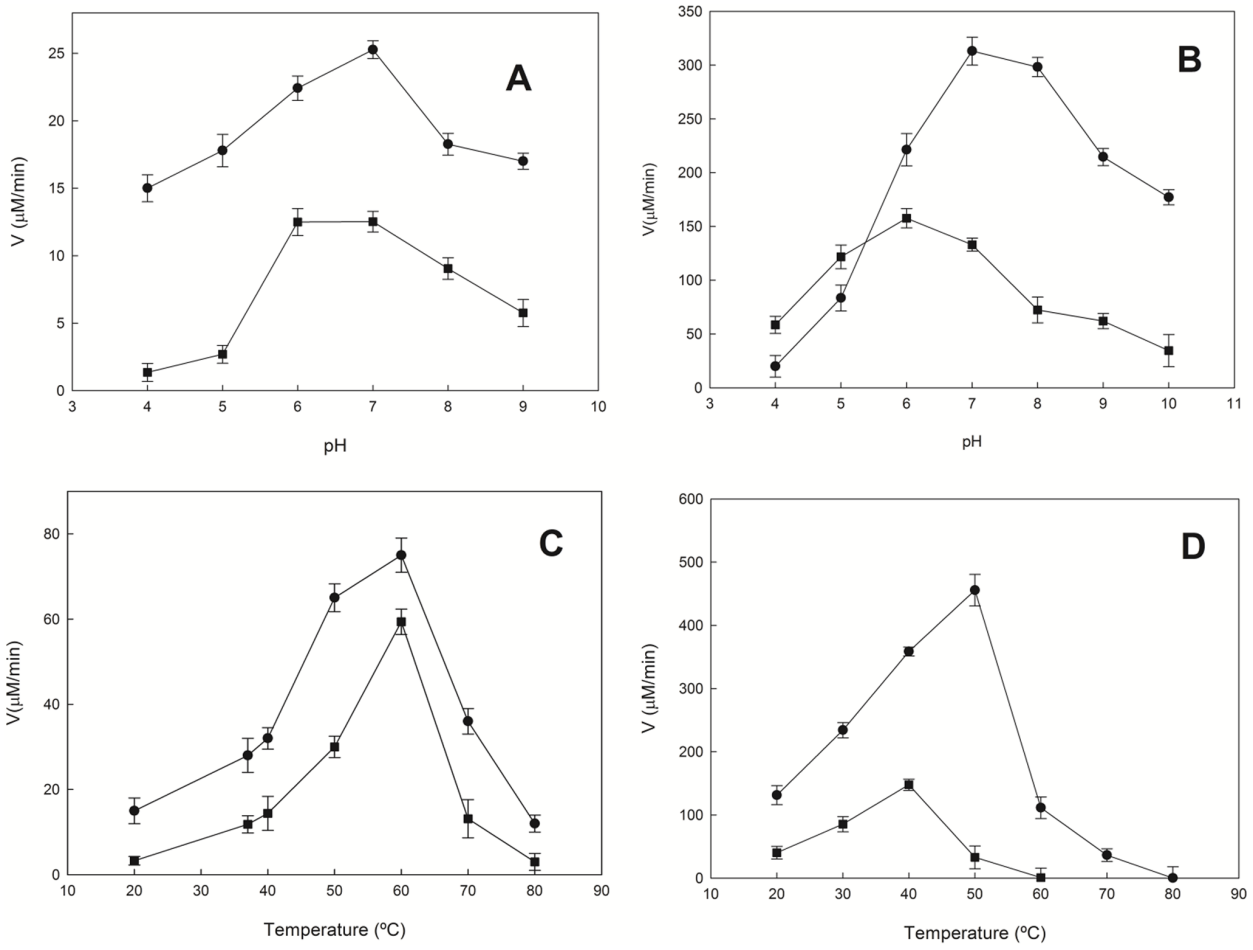
Effects of pH and temperature on the activity of LaNAL and LsNAL. A) Optimum pH of LaNAL for Neu5Ac synthesis (*▪*) and hydrolysis (*•*). Standard reaction conditions for HPLC at 37°C were used with the following buffers: 20 mM sodium acetate pH 4.5–5, sodium phosphate pH 6–7, Tris-HCl pH 8.5 and glycine pH 9. B) Optimum pH of LsNAL for Neu5Ac synthesis (*▪*) and hydrolysis (*•*). Assay conditions were the standard reaction medium for LaNAL. (C and D) Optimum temperature of LaNAL and LsNAL for Neu5Ac synthesis (▪) and hydrolysis (•). The activity was determined by HPLC at different temperatures under the corresponding standard reaction medium.

LaNAL was active from pH 4 to 9, showing different optimum pHs according to whether the hydrolytic or in the synthetic reaction was being followed. The maximum activity for Neu5Ac hydrolysis was found at pH 7.0 (Figure 2A, circles), whereas on the synthetic side, the maximum was found to lie between pH 6.0 to 7.0 (Figure 2A, squares). This shift to acidic pHs in the synthetic reaction was also observed in LsNAL (Figure 2B, squares), which was 1.5 pH units lower than NALs from groups 1 and 2 [8], [9], [13], [14], [27]. In addition, both LaNAL and LsNAL maintained about 45–50% of the activity in the synthetic direction at pH 9.0 (Figure 2A and B, squares). These values are lower than those previously described for NALs from groups 1 and 2 at this pH [8], [9], [13], [14], [27]. The differences in activity at basic pH with respect to groups 1 and 2 were more evident when their pH-stability was studied. LaNAL showed a sharp inactivation at pH 8.0–9.0 (Figure S2A), whereas LsNAL was somewhat more stable than LaNAL at these basic pHs (Figure S2B). This instability at basic pHs was reduced by incubating the enzyme in the presence of 5 mM pyruvate. The LaNAL half-life doubled at pH 8 (Figure S2A, filled circles), underlining the stabilizing power of this co-substrate, as previously described for other NALs [14].

In addition, LaNAL and LsNAL showed differences in their optimum temperatures compared with their counterparts from groups 1 and 2. The highest activity for LaNAL was found at 60°C, both in synthetic and hydrolytic reactions (Figure 2C). LsNAL showed a peak of activity at 50°C for aldol condensation and 40°C for its cleavage (Figure 2D). These values were clearly lower than those reported for the hydrolytic reaction in EcNAL (80°C) [8] and LpNAL (70°C) [13] but close to that reported for *Pasteurella multocida* (37°C) [9]. When thermostability was studied, LsNAL lost its activity within 1–2 hours, even at low temperatures (40–50°C) (Figure S3A). This rapid inactivation could be prevented in the presence of pyruvate, especially in the range 50–60°C (Figure S3B). This positive effect of pyruvate as regards both pH and temperature stabilization was independent of the well-documented competitive inhibition described for other NALs [8], [28], since the remaining pyruvate (0.5 mM ) in the reaction medium caused only a 7% and 4% inhibition in LaNAL and LsNAL, respectively. In fact, the values K_I_ for LaNAL and LsNAL were 0.16 mM and 0.4 mM, respectively (Figure S4). These K_I_ values were also different from those obtained for ManNAc, which were 157 mM for LaNAL and 114 mM for LsNAL (data not shown).

The low thermal stabilities of LaNAL and LsNAL were also evident when their protein melting temperatures (*Tm*) were calculated (Figure S5). Both enzymes showed a T*m* value of 50°C in Milli-Q water. In the presence of a buffer solution (100 mM phosphate buffer pH 7.0) this value increased to 65°C in LaNAL (Figure S5A), but decreased to 42°C in case of LsNAL (Figure S5B). When ΔT*m* (T*m* value obtained after subtracting Milli-Q water T*m*) was plotted *vs* pH for LsNAL (Figure S5, inset), the profiles obtained resembled those shown for synthetic activity in Figures 2A and 2B for both enzymes. Surprisingly, LsNAL was more stable at pH 5.0 than at its optimum pH of 6.0 (Figure S5B). This is a unique property that has never been described for other NALs [13], [14], but not so unusual in *L. sakei 23K*, where an acid-tolerant L-arabinose isomerase has been described with a broad an optimal pH (pH 5–7) and optimal temperature of 30–40°C [29]. In the presence of 5 mM pyruvate (Figure S5 inset, grey bars), the T*m* values also resembled the pH profiles shown in Figure 2, although the protective effect was more evident at basic pHs (Figure S5 inset, grey bars), especially in the case of LsNAL.

### LaNAL could be used as a biocatalyst for the production of antimicrobials

**Figure 3:**
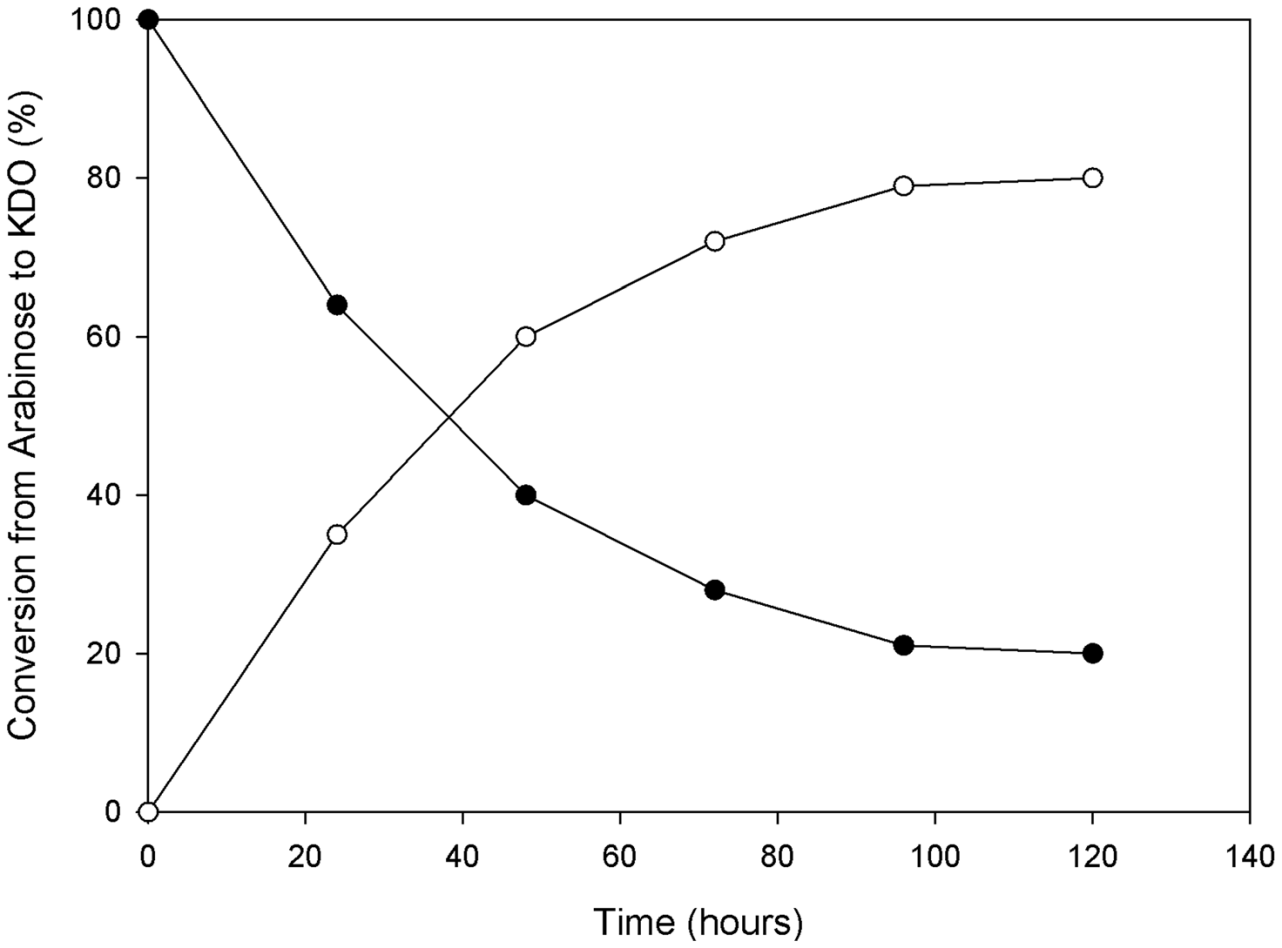
KDO synthesis from D-arabinose and pyruvate using LaNAL. Activity was assayed at 37°C with 0.6 M D-arabinose, 1.2 M pyruvate and 1 mg mL^−1^ LaNAL. D-arabinose (•) and KDO (○).

LaNAL preferentially used ManNAc as a substrate in aldol condensation with pyruvate (Table 1), although D-mannose also provided a measurable rate. NALs from groups 1 and 2 maintained nearly full activity with D-mannose, indicating the low specificity for the acetamide group, as revealed by the lack of interactions between this group, and the protein in the crystal structures available [10]. However, in the case of LaNAL, the activity with D-mannose decreased to 10%, indicating that the acetamide moiety in group 3 NALs plays a key role in substrate specificity unlike in its group 1 and 2 counterparts. In case of pentoses (D-arabinose and D-lyxose), the activity shown was lower than that observed with D-mannose, even though these two last sugars also fulfilled the precondition of having a 4-hydroxyl group to act as NAL substrate. The latter hydroxyl group interacts with the active site tyrosine (Tyr137 in EcNAL and Tyr139 in LaNAL), and plays a crucial role in the donation of a proton during the carbon-carbon formation between sugar and pyruvate [10], [30], [31]. Similar results as those described in Table 1 were described for EcNAL [32]. Interestingly, the relative activity for D-arabinose was equal to that of EcNAL and LpNAL (Table 1), opening up the possibility of using LaNAL as biocatalyst for the production of 3-deoxy-D-manno-2-octulosonic acid (D-KDO), an important synthon for antimicrobials towards the enzymatic Gram- cell wall assembly [30]. In fact, when LaNAL (1 mg mL^−1^) was used at 37°C in 20 mM phosphate buffer pH 7.0 with a 2∶1 pyruvate:D-arabinose ratio (1.2 M vs 0.6 M), 80% conversion into KDO was obtained in 120 hours (Figure 3). This conversion value was similar to that described for EcNAL (83%) [32], but the conditions were different, since in the latter case the conversion was obtained at a high concentration of the acceptor D-arabinose (ratio 1:25; i.e. 20 mM pyr: 500 mM D-arabinose) and with a higher enzyme concentration (8 mg mL^−1^).

**Table 1:** Substrate specificity of LaNAL.

Substrate or analog	Relative activity (%)		
	LaNAL†	LpNAL	EcNAL¶
**N-acetyl-D-mannosamine**	100	100	100
**D-Mannose**	10	95	91
**D-Arabinose**	1.5	1	1.2
**L-gulose**	0.7	3.6	20
**D-lyxose**	0.6	2.4	N.D.*
**2-deoxy-D-glucose**	1.2	33	35

† Reaction medium (100 µL) contained 0.6 M sugar, 1.2 M sodium pyruvate and 1 mg/mL purified LaNAL or LpNAL (13) in 20 mM phosphate pH 7.0. The activity was measured by HPLC/ELSD II.

¶ Taken from Lin *et al*., (29).

^*^N.D. Not Determined.

### High affinity for pyruvate and low affinity for ManNAc are key characteristics of LaNAL and LsNAL

Kinetic parameters were determined for the hydrolytic (Neu5Ac) and synthetic (ManNAc + pyr) reactions. The K_M_ for Neu5Ac cleavage was the lowest of those described (Table 2), with a value of 1.1 mM for LaNAL, and a surprisingly low 0.32 mM for LsNAL. These values were far from those previously described (1.8–4.5 mM) for groups 1 and 2 NALs [8], [9], [13], [14], [27]. The catalytic efficiency (*k_cat_*/K_M_) of both group 3 NALs (1.7 mM^−1^s^−1^ for LaNAL and 2.8 mM^−1^s^−1^ for LsNAL) was slightly lower than those previously reported for other NALs, which ranged from 5.6 to 2 mM^−1^s^−1^ [13], [14]. As regards synthesis, both enzymes showed higher K_M_ values for ManNAc (333 mM for LaNAL and 272 mM for LsNAL) compared with group 1 (149–220 mM) [13], [14] and group 2 (180 mM) [9]. However, their K_M_ values for pyruvate were the lowest described (1.1 mM for LaNAL and 12.5 mM for LsNAL), giving rise to the highest *k_cat_*/K_M_ values reported for pyruvate (5.1 mM^−1^s^−1^ for LaNAL and 0.31 mM^−1^s^−1^ for LsNAL) (Table 2), compared with the of 0.08 mM^−1^s^−1^ and 0.21 mM^−1^s^−1^reported for EcNAL [9] and ScNAL [14], respectively.

**Table 2:** Kinetic parameters of LsNAL, LaNAL and three mutants of LaNAL¶.

	LsNAL			LaNAL			LaNAL	LaNAL			LaNAL		
							G211S	G210S/Y213G			P193Y		
**Activities**	Cleavage	Synthesis		Cleavage	Synthesis		Cleavage	Cleavage	Synthesis		Cleavage	Synthesis	
**Substrate**	Neu5Ac	ManNAc	Pyr	Neu5Ac	ManNAc	Pyr	Neu5Ac	Neu5Ac	ManNAc	Pyr	Neu5Ac	ManNAc	Pyr
**K_M_ (mM)**	0.32±0.03	272±1	12.5±0.2	1.1±0.1	333±2	1.1±0.2	1.2±0.1	2.0±0.2	3200±120	4.3±02	0.27±0.02	915± 3	0.5±0.01
**k** ***_cat_*** ** (s^−1^)**	0.9±0.01	4.6±0.2	3.9±0.1	1.9±0.2	5.4±0.2	5.6±0.1	0.032±0.002	0.47±0.01	15.3±0.2	3.0±0.3	0.13±0.03	2.4±0.2	0.99±0.03
**k** ***_cat_*** **/K_M_ (mM^−1^s^−1^)**	2.8	0.016	0.31	1.7	0.016	5.1	0.026	0.23	0.004	0.71	0.48	0.002	1.98

¶ Data represent the mean values of three replicates with the SD indicated.

To further our knowledge of the kinetic characteristics, a mutational analysis was carried out with LaNAL (Table 2). The mutations were chosen based on two conserved blocks, one involved in sugar-binding (**G**[F/Y/P/V]**DE**) and the other in aldol-cleavage [I/V G/S S/G **T**) (Figure 1) [13]. The first mutant G211S, in which the second conserved glycine in the aldol-cleavage block is replaced by serine as in groups 1 and 2 NAL, showed a 100-fold decrease in catalytic efficiency to 0.028M^−1^s^−1^ (Table 2). This meant that it was impossible to detect any synthetic activity. The second mutant (G210S) analyzed the first glycine in the aldol-cleavage block, which is highly conserved in all phylogenetic groups, except in group 4.1, where it was replaced by a serine [13]. The NAL crystal structures [8], [10], [30], [31], [33] confirmed the conservation of this glycine because any other residue would restrict substrate binding. In fact, the *in silico* LaNAL model showed that this mutation clashes with Y213 (data not shown). Thus, a double mutant G210S/Y213G was constructed, showing a relatively inactive enzyme towards ManNAc (K_M_ = 3200 mM, *k*_cat_/K_M_ = 0.004 M^−1^s^−1^) and for pyruvate (K_M_ = 4.3 mM, *k*_cat_/K_M_ = 0.71 M^−1^s^−1^). The third mutant (P193Y), which resembled group 2 NAL sugar-binding motif, also showed a decrease in catalytic efficiency in both synthetic and hydrolytic reactions (Table 2), with a notable 3-fold increase in the K_M_ for ManNAc, up to 915 mM. Taking all the above kinetic data together, a plausible explanation of the low catalytic efficiencies of wild-type LaNAL and LsNAL could be related with the small size of their corresponding sugar-binding pockets (Figure S6), as was previously described for the crystal structures of RS-aldolase or L-KDO aldolase (an eight point mutation EcNAL) and several mutants of Val251 *E. coli* NAL [30]. These authors showed that constricting the sugar-binding pocket of EcNAL by point mutations at V251 produced a clear decrease in Neu5Ac cleavage activity, ranging from 60% (V251L) to 80% in a double mutant (V251L/V265L). This narrowing of the Neu5Ac-binding pocket was beneficial enabling it to accommodate the eight-carbon sugar KDO, giving rise to the highest ratio of cleavage activity for L-KDO and Neu5Ac in mutant V251W [30]. In our case, the possible reduction of the sugar-binding pocket by a larger residue at position G210 (G210S/Y213G) produced a clear increase in all K_M_ values and a concomitant decrease in all catalytic efficiencies (Table 2). This reduction in size could be more pronounced in mutant G211S, because very low hydrolytic activity towards Neu5Ac and no synthetic activity were detected. However, the effect on kinetic parameters of P193Y mutant could be related with a reduction in size of the active site pocket and the conformational changes induced in the neighboring residue E195, which is directly involved in the binding of the O8 and O9 atoms of Neu5Ac. In addition, the relatively good KDO synthetic activity of LaNAL could also be explained by the relatively small sugar-binding pocket, as previously described for Val251 EcNAL mutants [30]. In this respect, double mutant G210/Y213G was also able to convert five carbons sugars (D-mannose and D-arabinose) in the aldol condensation reaction into their corresponding eight carbon acids [2-keto-3-deoxy-d-glycero-galactononulosonic (KDN) and KDO], but a slower rate (up to ∼10% and ∼1% in seven days, respectively). These low conversions were in agreement with the low catalytic efficiency values shown by this double mutant in Table 2 and with the relative activity shown for the conversion of five carbons sugars by LaNAL (Table 1).

### Functional divergence analysis showed that group 3 NALs are more closely related to group 2 NALs

**Figure 4:**
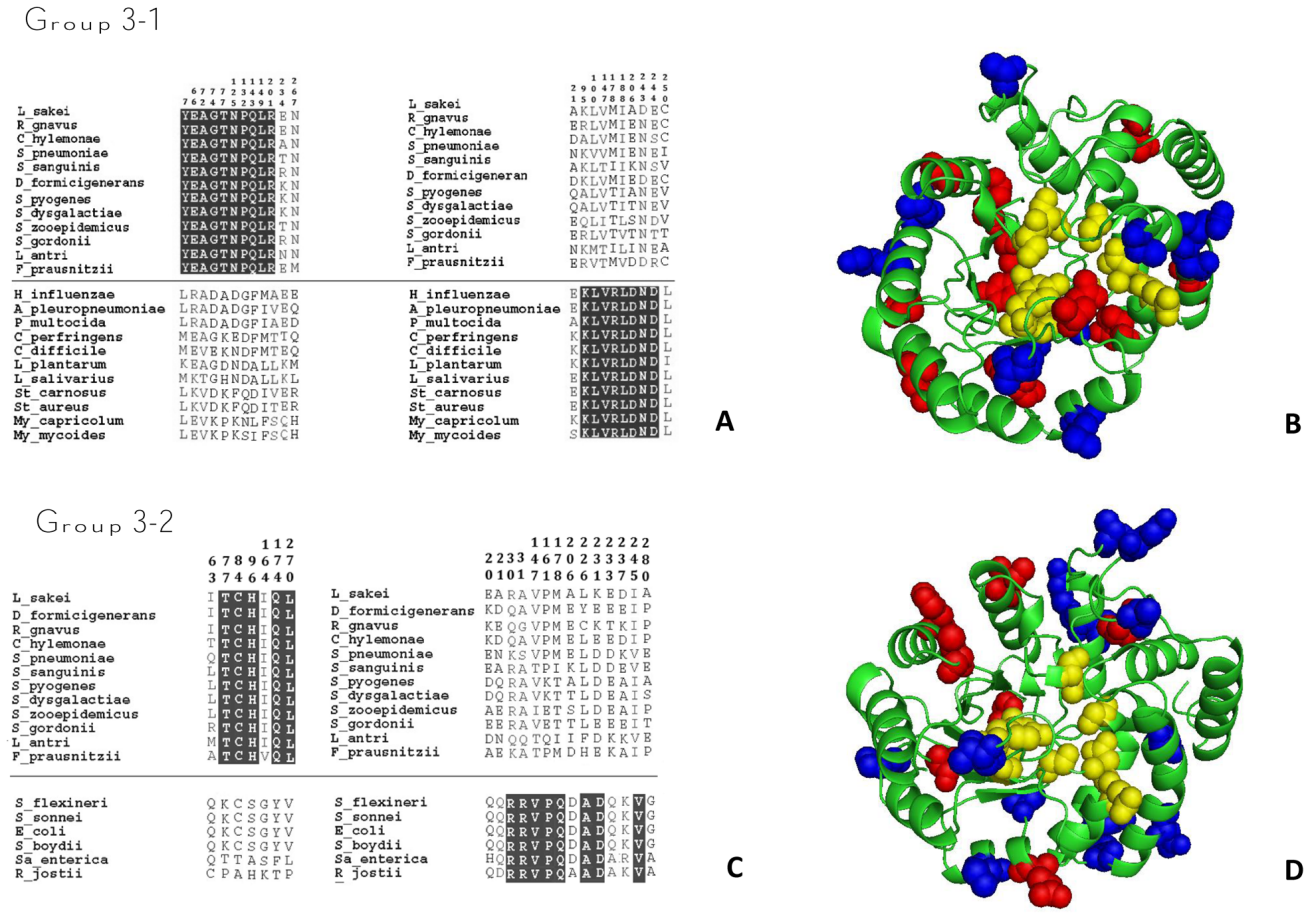
Functional divergence analysis of NALs. A) Analysis of group 3 and 1. B) Molecular representation of Cat I and Cat II residues in group 3 and group 1. C) Analysis of group 3 and 2. D) Molecular representation of Cat I and Cat II residues of group 3 and 2. The residues forming the active site are indicated with yellow spheres, those forming the Category I are indicated by red spheres and those forming Category II by blue spheres.

To further characterize the differences between group 3 NALs and NALs from groups 1 and 2, a functional divergence analysis was carried out to detect amino acid sites that have varying evolutionary conservation statutes among NAL members, using DIVERGE (Detecting Variability in Evolutionary Rates among Genes) software [26]. The analysis grouped the amino acids residues responsible for altered functional constraints into two categories: (I) conserved in the first group, but variable in the second group; (II) conserved in the second group, but variable in the first group. A site-specific profile based on probability (Q_k_) was used to identity critical amino acids [26], with a Q_k_ >0.75 (Figure 4). In fact, when group 3 and group 1 were compared, 10 amino acids (YEAGTNPQLR) were conserved in category I and eight in category II (KLVRLDND) (Figure 4A). This divergence was more pronounced than that found between group 3 and group 2, where only 5 amino acids (TCHQL) were conserved in category I and eight in category II (Figure 4C). This indicates, as mentioned above, that group 2 and group 3 are more closely related.

To visualize these divergence sites, a 3D representation was made for each pair of groups (3→1 and 3→2) (Figures 4B and 4D, respectively). It was clear that divergent amino acids were basically in the outer helices (Figures 4B and 4D, red spheres are Cat.I, blue spheres are Cat. II) or close to the internal β-barrel, but did not affecting the active site (Figure 4, yellow spheres). This clearly indicates that the drift at these sites was well tolerated, with no loss of activity. These changes were outside the conserved blocks related with catalysis (G_48_SSGE, K_167_NSS, G_192_PDE and I_209_GGT), and were basically located between helices α_2_–α_3_ and α_4_–α_6_, respectively (Figure S7).

## Conclusions

This paper describes, for the first time, the intrinsic characteristics of two representatives of NALs from group 3, LaNAL and LsNAL, which showed differences from those of group 1 and 2, not only under a phylogenetic point of view but also biochemically. These differences included lower optimum pHs and lower stability. In addition, LaNAL and LsNAL showed a high K_M_ for ManNAc and very low K_M_ for pyruvate. These differences can be related with the presence a small sugar-binding pocket in these two NALs. Future studies with other group 3 and group 4 NALs are needed to provide new insights into their biology, biochemistry and evolution, which are related to the remarkably common and diverge Neu5Ac, found in the contact surface between many bacteria and their external environmental, including the human body.

## Supporting Information

Figure S1 **SDS-PAGE analysis of LaNAL in the different purification steps.** Lane 1: crude extract after DNase treatment. Lane 2: crude extract after 100-kDa tangential ultrafiltration. Lane 3: purified LaNAL (35 kDa) after HisTrap column. Each lane contained 15 µg of protein. M: molecular weight standards (New England Biolabs: P7708S).
(TIFF)

Figure S2 **Effect of pH on LaNAL and LsNAL stability.** A) LaNAL was incubated at 37°C in different buffers of pH 6.0 (□) pH 7.0 (▪), pH 8.0 (○) and pH 9.0 (▴), and samples were taken after the indicated periods for assays of enzyme activity. The activity was measured spectrophotometrycally under the standard reaction conditions at pH 7.0 using Neu5Ac as substrate. Also, the pH-stability of the purified LaNAL incubated at pH 8.0 in the presence of 5 mM sodium pyruvate is shown (•), the activity was measured spectrophotometrically using ManNAc dehydrogenase as described in Material and Methods. B) LsNAL was incubated at 37°C for the indicated periods at pH 6.0 (□) pH 7.0 (▪), pH 8.0 (○) and pH 9.0 (▴), and the activity was measured spectrophotometrically under its corresponding standard conditions.
(TIFF)

Figure S3 **Effect of temperature on the LsNAL stability.** A) The enzyme was incubated at 40°C (•), 50°C (○), 60°C (▪) and 70°C (□) at pH 7.0, and samples were taken after the indicated periods for enzyme activity assays. The activity was measured spectrophotometrically under the standard reaction conditions at 37°C using Neu5Ac as substrate. B) Thermal inactivation of LsNAL. The enzyme was incubated at the indicated temperatures for 10 min in 20 mM sodium phosphate buffer (pH 7.0) in the absence (•) and presence of 5 mM sodium pyruvate (○). The activity was measured spectrophotometrically using ManNAc dehydrogenase as described in Material and Methods.
(TIFF)

Figure S4 **Inhibition of LaNAL and LsNAL by pyruvate.** (A) Double-reciprocal plot of LaNAL activity in the presence of different pyruvate concentrations: 0 mM (○), 0.5 mM (▪), 1.5 mM (□) and 5 mM (•). The activity was measured spectrophotometrically using the enzyme-coupled assay with ManNAc dehydrogenase. Inset. Secondary plot of K_M_^app^ as a function of pyruvate concentration. The K_I_ value was determined from the abscissa intercept. (B) Double-reciprocal plot of LsNAL activity in the presence of different pyruvate concentrations. The conditions are the same as in (A). Inset. Inset. Secondary plot of K_M_^app^ as a function of pyruvate concentration.
(TIF)

Figure S5 **Melting curves of LaNAL and LsNAL.** (A) LaNAL and (B) LsNAL unfolding was monitored with SYPRO Orange dye with 1 µg of purified enzyme. Curves were obtained in MilliQ water (○) and in the presence of the buffers described in Figure 2: pH 4 (▴), pH 5 (◊), pH 6 (▪), pH 7 (□), pH 8 (•) and pH 9 (▾). Inset**.** Effect of pyruvate on melting temperatures of LaNAL and LsNAL at different pHs. Black and light grey bars represent absence or presence of 5 mM sodium pyruvate, respectively. Assays were performed in a real time PCR apparatus with 10X SYPRO Orange.
(TIFF)

Figure S6 **Active site comparison of NALs from groups 1, 2 and 3 (LaNAL and LsNAL).** The active site of sialic acid alditol for: (A) HiNAL (group 1, PDB ID 1F73) and (B) EcNAL (group 2, PDB ID 1NAL) are compared with the models of group 3 NALs from (C) LaNAL and (D) LsNAL. The sialic acid alditol was modelled from PDB code 1F73. Residues involved in substrate binding are represented by balls and sticks. Residues subjected to mutational analysis are in yellow. The interactions with the C6 and C7 atoms of sialic acid are shown.
(TIF)

Figure S7 **Multiple sequence alignment of N-acetylneuraminate lyases from group 3, indicating position of Cat I and Cat II residues.** The background of residues strictly conserved across NAL enzymes is filled. The secondary structure of LsNAL is shown: springs represent helices and arrows represent β-stands. Residues belonging to the active site are indicated by squares. Residues forming Category I in groups 3 and 1 are indicated by triangles up, the residues forming Category II in groups 3 and 1 are indicated by triangles down, the residues forming Category I in groups 3 and 2 are indicated by filled circles, the residues forming Category II in groups 3 and 2 are indicated by open circles, the common Category II residues in groups 3 and 1 and in groups 3 and 2 are indicated by stars.
(TIFF)

Table S1 **Oligonucleotide primers used for LaNAL site-directed mutagenesis.**
(PDF)

Table S2 **Purification of recombinant LaNAL.**
(PDF)
