# Supplementary figures and images for: First Functional and Mutational Analysis of Group 3 N-Acetylneuraminate Lyases from Lactobacillus antri and Lactobacillus sakei 23K
Source: PLoS One. 2014 May 9;9(5):e96976. doi: 10.1371/journal.pone.0096976 (PMC4016182; doi:10.1371/journal.pone.0096976)

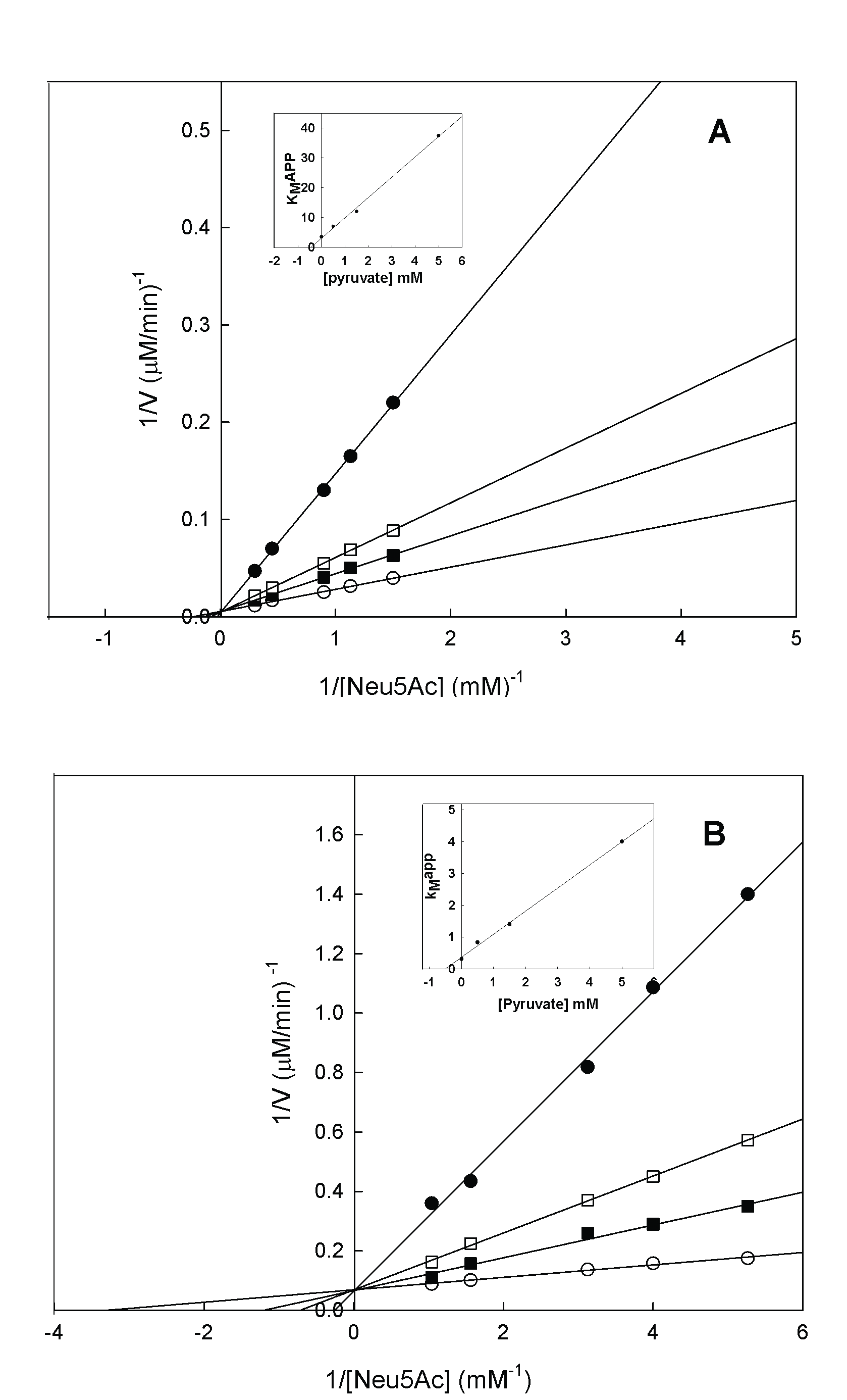

Supplement: Figure S4 — Inhibition of LaNAL and LsNAL by pyruvate. (A) Double-reciprocal plot of LaNAL activity in the presence of different pyruvate concentrations: 0 mM (○), 0.5 mM (▪), 1.5 mM (□) and 5 mM (•). The activity was measured spectrophotometrically using the enzyme-coupled assay with ManNAc dehydrogenase. Inset. Secondary plot of KM app as a function of pyruvate concentration. The KI value was determined from the abscissa intercept. (B) Double-reciprocal plot of LsNAL activity in the presence of different pyruvate concentrations. The conditions are the same as in (A). Inset. Inset. Secondary plot of KM app as a function of pyruvate concentration. (TIF) [file pone.0096976.s004.tif]

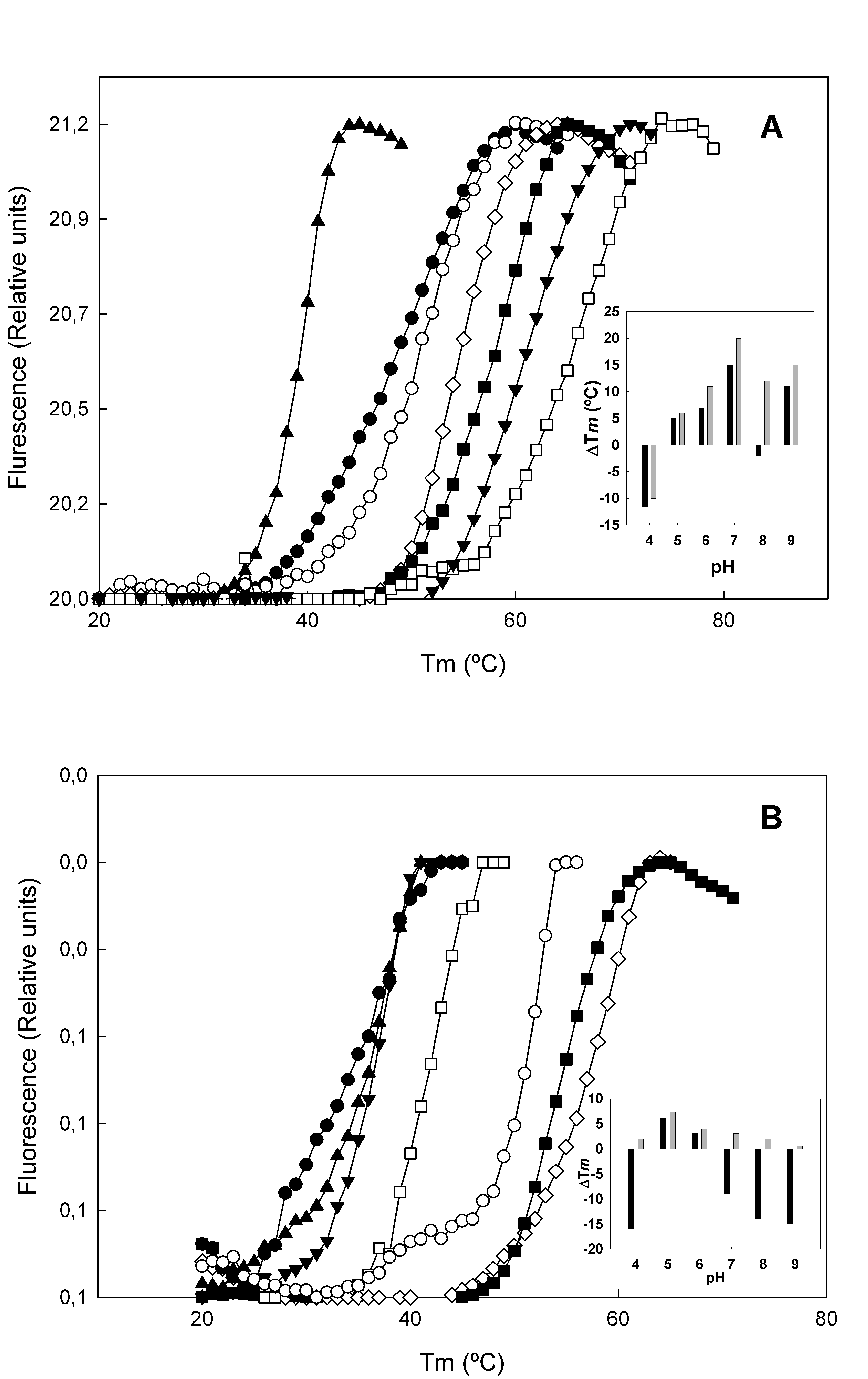

Supplement: Figure S5 — Melting curves of LaNAL and LsNAL. (A) LaNAL and (B) LsNAL unfolding was monitored with SYPRO Orange dye with 1 µg of purified enzyme. Curves were obtained in MilliQ water (○) and in the presence of the buffers described in Figure 2: pH 4 (▴), pH 5 (◊), pH 6 (▪), pH 7 (□), pH 8 (•) and pH 9 (▾). Inset. Effect of pyruvate on melting temperatures of LaNAL and LsNAL at different pHs. Black and light grey bars represent absence or presence of 5 mM sodium pyruvate, respectively. Assays were performed in a real time PCR apparatus with 10X SYPRO Orange. (TIFF) [file pone.0096976.s005.tiff]

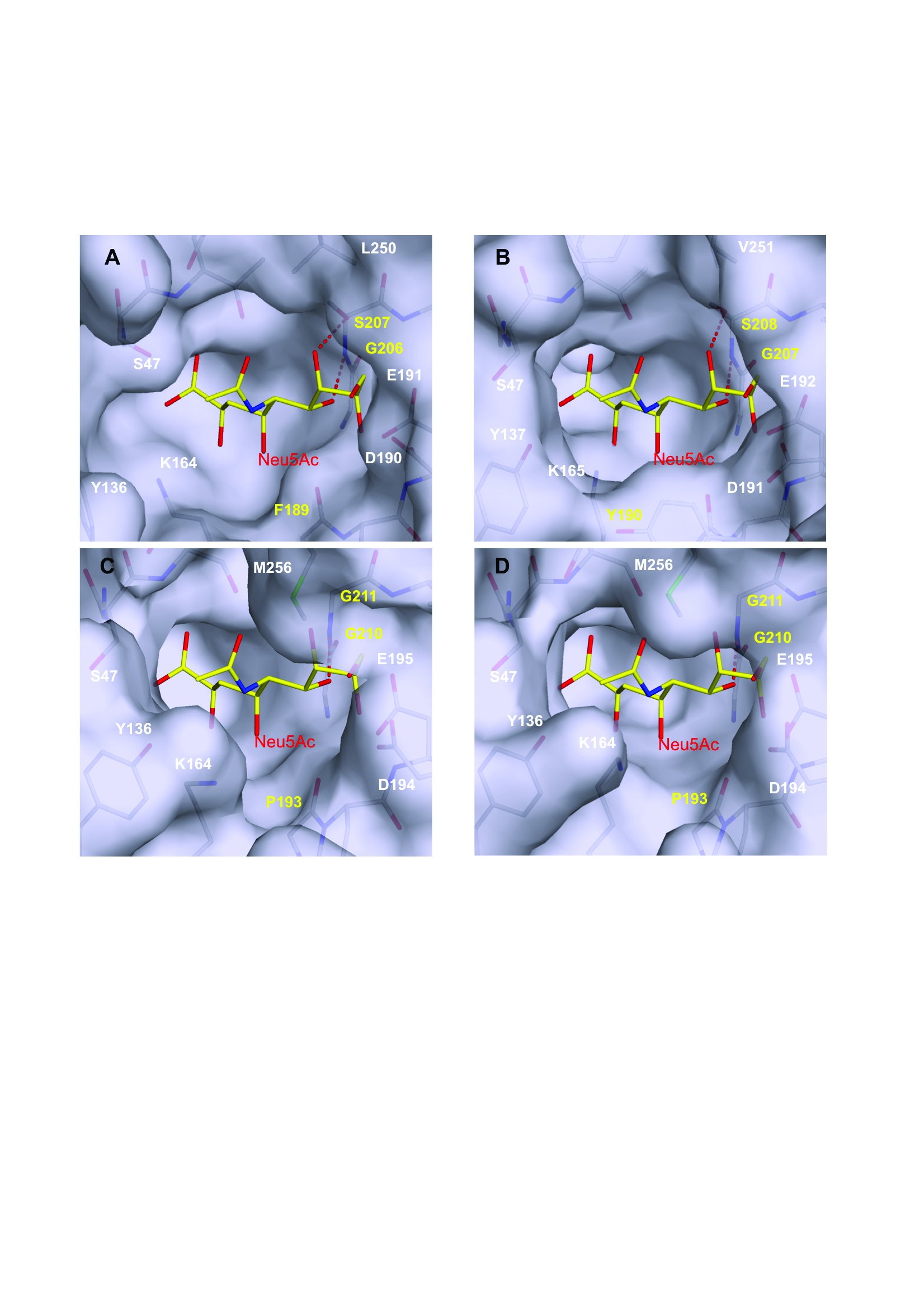

Supplement: Figure S6 — Active site comparison of NALs from groups 1, 2 and 3 (LaNAL and LsNAL). The active site of sialic acid alditol for: (A) HiNAL (group 1, PDB ID 1F73) and (B) EcNAL (group 2, PDB ID 1NAL) are compared with the models of group 3 NALs from (C) LaNAL and (D) LsNAL. The sialic acid alditol was modelled from PDB code 1F73. Residues involved in substrate binding are represented by balls and sticks. Residues subjected to mutational analysis are in yellow. The interactions with the C6 and C7 atoms of sialic acid are shown. (TIF) [file pone.0096976.s006.tif]

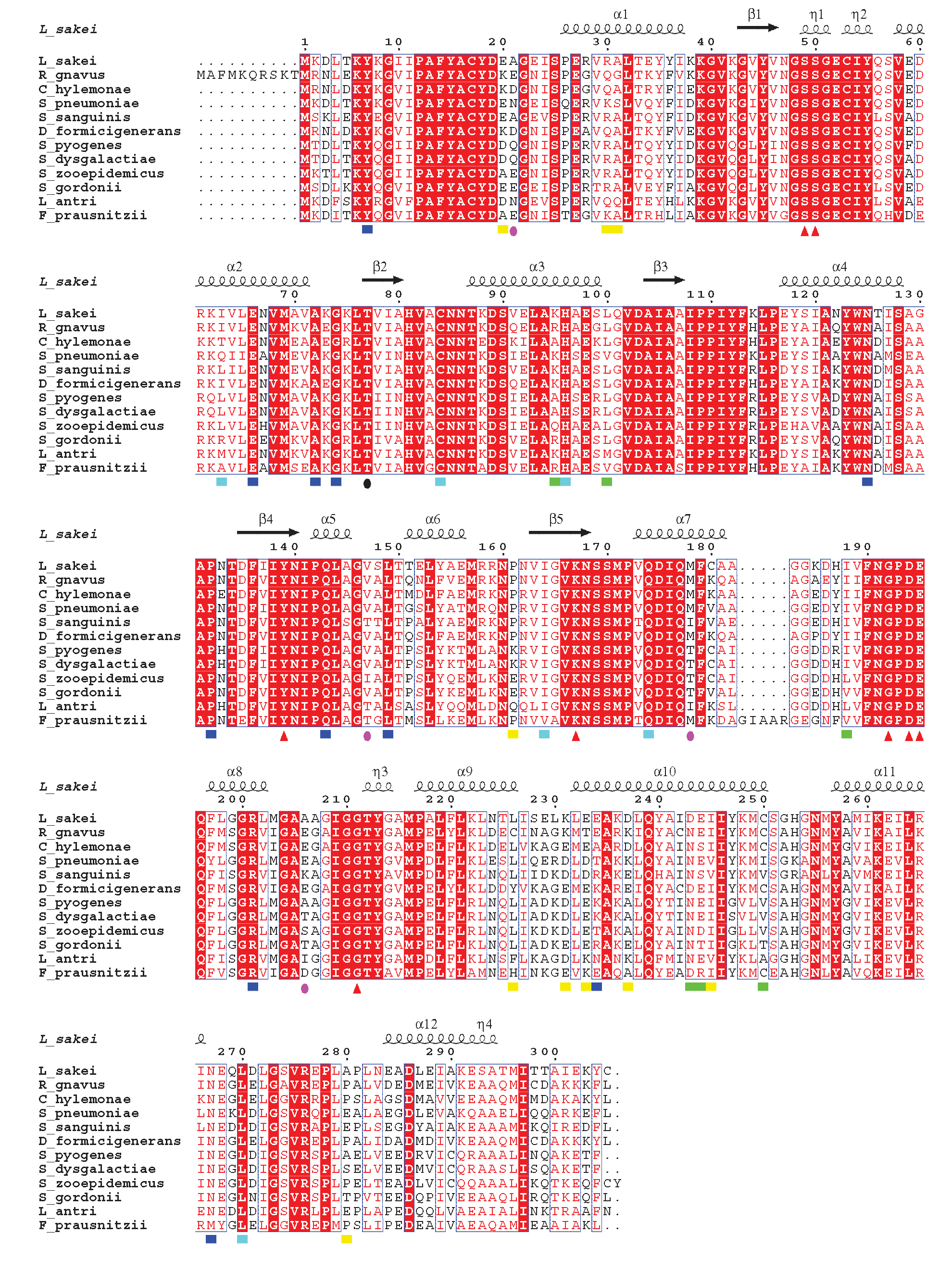

Supplement: Figure S7 — Multiple sequence alignment of N-acetylneuraminate lyases from group 3, indicating position of Cat I and Cat II residues. The background of residues strictly conserved across NAL enzymes is filled. The secondary structure of LsNAL is shown: springs represent helices and arrows represent β-stands. Residues belonging to the active site are indicated by squares. Residues forming Category I in groups 3 and 1 are indicated by triangles up, the residues forming Category II in groups 3 and 1 are indicated by triangles down, the residues forming Category I in groups 3 and 2 are indicated by filled circles, the residues forming Category II in groups 3 and 2 are indicated by open circles, the common Category II residues in groups 3 and 1 and in groups 3 and 2 are indicated by stars. (TIFF) [file pone.0096976.s007.tiff]
