# Supplementary material for: First Functional and Mutational Analysis of Group 3 N-Acetylneuraminate Lyases from Lactobacillus antri and Lactobacillus sakei 23K
Source: PLoS One. 2014 May 9;9(5):e96976. doi: 10.1371/journal.pone.0096976 (PMC4016182; doi:10.1371/journal.pone.0096976)
Supplement: Table S1 — Oligonucleotide primers used for LaNAL site-directed mutagenesis. (PDF) [file pone.0096976.s008.pdf]

Table S1 Oligonucleotide primers used for LaNAL site-directed mutagenesis.

Primer name	Sequence (5`to 3´)[a]	Corresponding position in *EcNAL*
G211S-Forward	ATTGGCGGGATTGGTAGTACTTATGGTGCCATG	S208
G211S-Reverse	CATGGCACCATAAGTACTACCAATCCCGCCAAT	S208
P193Y-Forward	CCTGGTCTTTAATGGTTATGACGAGCAGTTTAT	Y190
P193Y-Reverse	ATAAACTGCTCGTCATAACCATTAAAGACCAGG	Y190
G210S-Y213G Forward	GCAATTGGCGGGATTAGTGGTACTGGTGGTGCCATGCC<br>CG	S207, Y210
G210S-Y213G Reverse	CGGGCATGGCACCACCAGTACCACTAATCCCGCCAATT<br>GC	S207, Y210

[a] Mutated bases are underlined.
