# Supplementary material for: First Functional and Mutational Analysis of Group 3 N-Acetylneuraminate Lyases from Lactobacillus antri and Lactobacillus sakei 23K
Source: PLoS One. 2014 May 9;9(5):e96976. doi: 10.1371/journal.pone.0096976 (PMC4016182; doi:10.1371/journal.pone.0096976)
Supplement: Table S2 — Purification of recombinant LaNAL. (PDF) [file pone.0096976.s009.pdf]

Table S2 Purification of recombinant LaNAL.

Purification Step	Volume (mL)	Total Activity (U)	Total protein (mg)	Specific activity (U mg^{-1})	Purification factor	Yield (%)
**Crude extract**[†]	990	6848	3217	2.1	1	100
**100 kDa Ultrafiltration**	220	5314	1762	3.0	1.4	77.6
**HiPrep IMAC**	324	5137	631	8.1	2.6	75.0

[†]Crude extract represents the volume obtained after lysis and centrifugation of cell debris and DNase treatment corresponding to a 4 L culture broth. See Material and Methods for details.
